# Thermodynamic limits of the depolymerization of poly(olefin)s using mechanochemistry†

**DOI:** 10.1039/d4mr00079j

**Published:** 2024-08-26

**Authors:** Yuchen Chang, Van Son Nguyen, Adrian H. Hergesell, Claire L. Seitzinger, Jan Meisner, Ina Vollmer, F. Joseph Schork, Carsten Sievers

**Affiliations:** a School of Chemical & Biomolecular Engineering, Georgia Institute of Technology Atlanta Georgia 30332 USA carsten.sievers@chbe.gatech.edu; b Inorganic Chemistry and Catalysis, Institute for Sustainable and Circular Chemistry, Utrecht University Universiteitsweg 99 3584 CG Utrecht The Netherlands; c Institute for Physical Chemistry, Heinrich Heine University Düsseldorf Universitätsstr 1 40225 Düsseldorf Germany

## Abstract

Mechanochemistry is a promising approach for chemical recycling of commodity plastics, and in some cases depolymerization to the monomer(s) has been reported. However, while poly(olefin)s comprise the largest share of global commodity plastics, mechanochemical depolymerization of these polymers in standard laboratory-scale ball mill reactors suffers from slow rates. In this work, the observed reactivities of poly(styrene), poly(ethylene) and poly(propylene) are rationalized on the basis of thermodynamic limitations of their depolymerization by depropagation of free radical intermediates. In addition, subsequent phase partitioning equilibria for the removal of monomers from the reactor *via* a purge gas stream are discussed for these polymers. For poly(styrene), a typical vibratory ball mill supplies just enough energy for its depolymerization to be driven by either thermal hotspots or adiabatic compression of the impact site, but the same energy supply is far from sufficient for poly(propylene) and poly(ethylene). Meanwhile, removal of styrene from the reactor is thermodynamically hindered by its lower volatility, but this is not an issue for either propylene or ethylene. The implications of these thermodynamic limitations for mechanochemical reactor design and potential for mechanocatalytic processes are highlighted.

## Abstract 2

Feasibility of mechanochemical depolymerization of commodity poly(olefin)s in a ball mill reactor is assessed using thermodynamic data.

## Graphical abstract

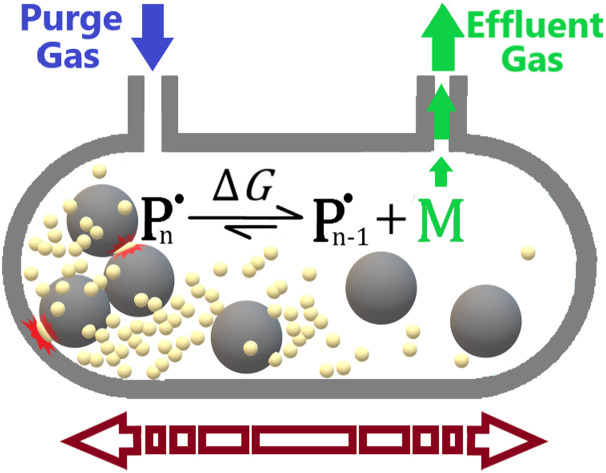

Plastic waste remains a persistent environmental issue in the present age,^1^ and novel chemical recycling processes are crucial in aiding the transition of commodity plastic materials towards greater environmental sustainability.^2,3^ Chemical recycling aims to convert waste plastics to other economically valuable chemical feedstocks, and depolymerization specifically aims to convert plastics back to their constituent monomers.^4^ The potential of mechanochemistry for chemical recycling of plastics has gained consideration due to the advantages it offers over liquid and solution-based alternatives, which include being able to process waste feedstocks in the solid state^5–7^ and greater flexibility in reactor design and scaling.^8^ Mechanochemical depolymerization of the polyester poly(ethylene terephthalate) (PET) has been demonstrated to achieve complete conversion of PET to its monomers using ball mill reactors, in a solid state reaction with sodium hydroxide,^9–11^ and mechanochemical methanolysis has also been demonstrated to achieve high yields for the polycarbonate Bisphenol A and poly(lactic acid).^12^

Ball mill reactors consist of loose macroscopic grinding bodies and solid reactant powders inside a mechanically agitated (shaking, rotation, other forms of periodic motion) vessel.^13–16^ Collisions and sustained mechanical contacts between grinding bodies transiently crush and compact the solid reactant powders in between their surfaces, a small portion at a time, and the compression and shearing forces experienced by the particles during compaction lead to enhanced solid–solid mixing, distortion of chemical bonds, thermal hot spots, and a variety of surface chemical phenomena resulting in solid state chemical reactions.^17–22^

Poly(olefin)s comprise the greatest share of commodity plastics production and waste generation.^4^ Compared to condensation polymers, the conversion of poly(olefin)s is more challenging due to the lack of labile bonds in the backbone of these polymers, but proof-of-concept mechanochemical approaches utilizing a ball mill reactor have appeared for poly(styrene) (PS),^23^ poly(propylene) (PP),^24^ poly(ethylene) (PE),^25^ and poly(methyl methacrylate) (PMMA).^26^ More detailed studies of mechanochemical depolymerization kinetics have been undertaken by Chang *et al.*^27^ for PS and by Jung *et al.* for its structural derivative poly(α-methylstyrene) (PMS)^28^ in vibratory ball mills. PMS – which is highly depolymerizable on account of its low ceiling temperature – was found to convert to an asymptotic amount of monomer in a sealed reactor which increases with mill frequency, but the achievable conversion fell short of 100%.^28^ PS exhibited much slower kinetics, being produced at an approximately constant rate on the order of milligrams per gram of PS per hour, but if monomer was not removed continuously from the reactor by flowing a purge gas stream through the reactor during milling, repolymerization becomes an issue when the amount of styrene in the reactor has accumulated to tens of milligrams per gram of PS feed.^27^

These observations raise the important question of whether the formation of monomers by mechanochemical depolymerization is limited by kinetic or thermodynamic constraints. This communication analyzes the depolymerization thermodynamics of the three commodity poly(olefin)s with the highest production volumes^4^ – PE, PP and PS – to their monomers *via* a mechanochemical mechanism to construct several thought experiments that demonstrate the range of thermodynamic feasibility with implications for engineering improvements to the process.

In mechanochemical reactors such as a vibratory ball mill, mechanochemical depolymerization events are created when grinding bodies (reactor wall and balls) collide due to mechanical agitation of the reactor which crush small quantities of solid polymer powder in between their surfaces.^10^ Therefore, the physical system we shall analyze – illustrated in Fig. 1 – consists of macroscopic grinding surfaces divided between the reactor interior wall (W) and grinding balls (B), and microscopic solid polymer particles (P), which are associated with average steady state surface temperatures *T*_W_, *T*_B_ ≈ *T*_W_, and *T*_P_, respectively. All space that is not occupied by these solid bodies is filled by a constant composition gas phase (G) at temperature *T*_G_ and pressure *p*_0_. In the subsequent discussion, this gas phase is taken to be pure nitrogen to reflect reported experimental conditions.^27^ When a polymer particle is crushed between two grinding surfaces, mechanochemical processes occur which can lead to the production of monomers (illustrated as light blue blotches in Fig. 1) during the course of the impact. A detailed kinetic study on PS^27^ observed a constant rate of monomer production for several hours of milling, with monomers exiting the reactor as vapor in the gas stream. On account of this, we postulate that mechanochemical styrene production from PS shares mechanistic similarities with mechanochemical reactions of gases,^29^ in the sense that most of the monomer production during impacts on the polymer particles occurs near the particle surfaces,^30^ with depolymerization instigated by surface chemical mechanisms such as particle fracture and microscopic friction between solid surfaces.^31^ In between impact events, monomers may freely volatilize into the gas phase in accordance with observations.

**Fig. 1:**
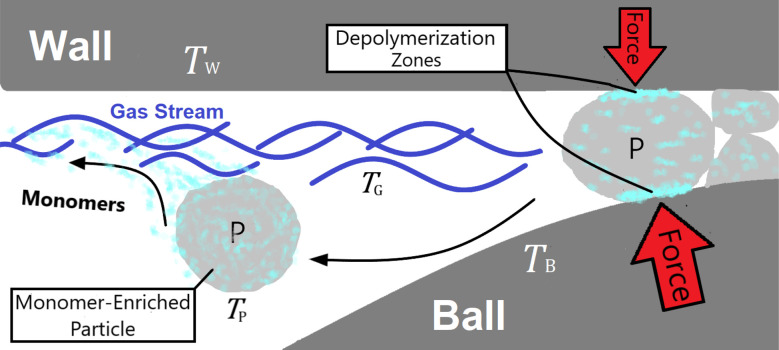
Model of the physical system. ‘W’ and ‘B’ denote reactor wall and ball respectively, ‘P’ denotes polymer particle and ‘G’ denotes gas phase.

The system depicted in Fig. 1 is regarded as a steady state system at the reactor time scale (on the order of hours). From previous studies of ball-milled PS,^27,32^ it is known that ball milling resulted in a rapid decrease of the average molecular weight (*M*_W_) of residual PS that tapers off at around 10 000 g mol^−1^ within two hours,^33^ while an approximately constant rate of monomer production was observed way past this point, which indicates that mechanochemical chain cleavage is not the only way to trigger and sustain monomer production.^27^ Using accepted radical mechanisms,^34–37^ at least five elementary steps are required to explain the reaction network of styrene depolymerization along with the simultaneous progression of *M*_W_ (Fig. 2). A nearly constant monomer production suggests steady state conditions with respect to the radical concentrations generated through reaction 1, sustaining a consistent pool of radicals so long as grinding persisted. Reaction 1 is primarily responsible for *M*_W_ degradation, but this mechanism becomes less relevant once the limiting *M*_W_ of 10 000 g mol^−1^ is attained. Depropagation (reaction 3) and propagation (reaction 4) are directly relevant to the production of monomers. The assumption of quasi steady conditions with respect to monomer production implies that a reservoir of active radicals generated through reaction 1 is continuously available, with separate mechanochemical events being responsible for advancing reactions 1 and 3. The occurrence of reactions 2 and 5 does lead to radical losses, but the rates of these steps are assumed to be balanced with reaction 1 in the steady state regime. This allows us to analyze how the local reaction environment created in a mechanochemical reactor determines the thermodynamic viability of reactions 3 and 4.

**Fig. 2:**
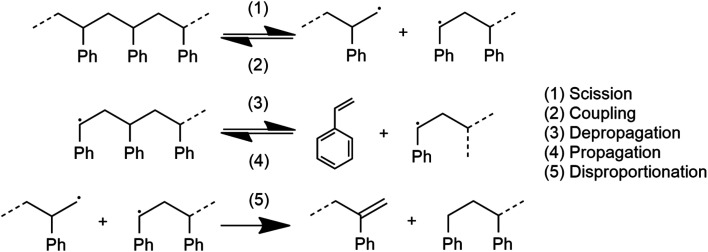
Elementary steps required in the mechanochemical depolymerization of poly(styrene).

The description advanced so far is based on experimental results of PS, but in subsequent discussion we shall apply the same thermodynamic analysis to PS, PP and PE. In the case of the latter two poly(olefin)s, reduced relative stability of their chain end radicals would lead to significantly more frequent instances of radical transfer reactions following scission (reaction 1) that form live midchain radicals;^38^ depropagation might not proceed from such midchain radicals in an analogous manner to PS. However, for the sake of comparison, in this study we shall simply assume a steady concentration of chain end radicals as a precondition of the analysis, with the aim of comparing thermodynamic characteristics of the three polymers with respect to the depropagation–propagation equilibrium.

For the physical mechanism of a depolymerization event, we adopt an idea proposed by Carta *et al.*:^39^ when the particles are subject to mechanical impact, mechanochemical reactions of solid particles occur predominantly in small pockets of “activated” volumes. Adapting this model to mechanochemical depolymerization, we claim that whenever a group of polymer particles is impacted in between two colliding grinding surfaces, a transient spell of depropagation occurs at microscopic regions on these particles. The thermodynamic viability of monomer production can be assessed based on the equilibrium of a single propagation–depropagation reaction step on a chain of *n* monomers and its associated equilibrium constant *K*_*n*_:1
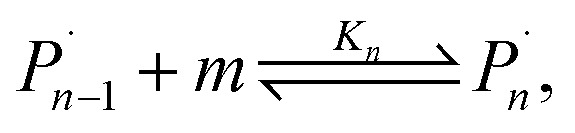
where *m* denotes the monomer species. Thermodynamically, we characterize the depropagation events by a molar Gibbs free energy of polymerization Δ_r_*G via*:

2 Δ_r_*G* = Δ_r_*H* − *T*Δ_r_*S*,

where Δ_r_*H* and Δ_r_*S* are the enthalpy and entropy of polymerization respectively, and *T* ≥ *T*_W_ is the temperature at which the transient depolymerization occurs.

The energies and entropy in eqn (2) are not standard condition values so they are themselves functions of *T*. However, for simplicity we assume the condition of standard pressure *p* = 101 325 Pa for all thermodynamic functions. To evaluate Δ_r_*G*, we make use of thermodynamic functions relating the molar enthalpy Δ_r_*H* and the molar entropy Δ_r_*S* of polymerization to the isobaric heat capacity of the reaction Δ_r_*C*:3
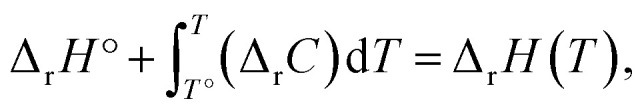
4
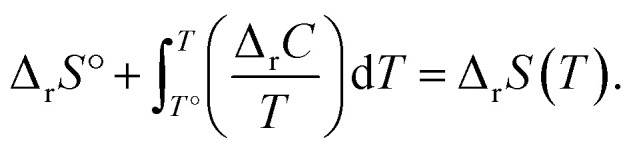


We assume that depropagation reactions convert polymer within a microscopic activated region to gaseous monomers, thus we can access Δ_r_*C* by taking the difference between the isobaric heat capacities of the pure (solid) polymer *C*_p_ and the pure monomer *C*_m_ in the gas state:

5 Δ_r_*C* = *C*_p_ − *C*_m_.

Δ_r_*H*°, Δ_r_*S*°, *C*_p_(*T*) and *C*_m_(*T*) are available in the literature for PS, PP and PE as well as for their monomers. The sources of thermodynamic data are summarized in Table 1.

**Table 1:** Literature references for thermodynamic data used in this work

Monomer	Ethylene	Propylene	Styrene
Sources for *C*_m_	NIST		
*T* range (K) for *C*_m_	298–1200	50–1200	50–1200
Source for *C*_p_	Wunderlich *et al.*^40^	Gaur & Wunderlich^41^	Gaur & Wunderlich^42^
*T* range (K) for *C*_p_	0–500	0–600	0–600
Source for Δ_r_*H*° and Δ_r_*S*°	Dainton & Ivin^43^		

The relation between eqn (2) and *K*_*n*_ associated with eqn (1) is according to the standard definition of the equilibrium constant, which can also be expressed as a ratio of species activities:6
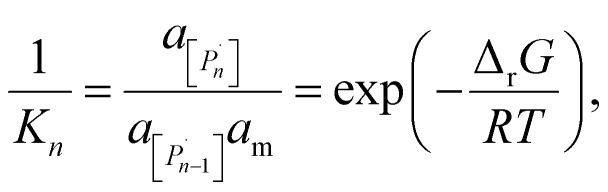
where *R* is the gas constant, *a*_m_ is the monomer activity, and the other two activities denote those of the polymer chain end radicals that differ by one monomer unit. Note that *K*_*n*_ is the equilibrium constant with respect to depropagation as the forward reaction, whereas Δ_r_*G* is the free energy with respect to propagation (as commonly tabulated in literature). For long chains, *K*_*n*_ is practically independent of *n*.^36^ Thus, there should be no difference in activity between reactive chain end radicals belonging to chains of different lengths,^44^ so:7
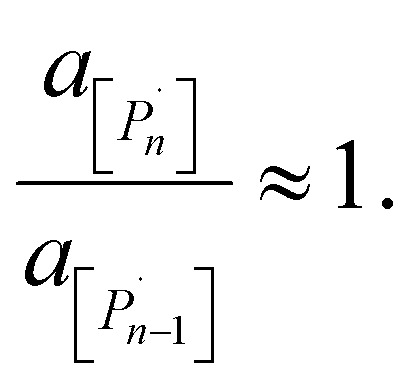


As an example of the applicability of eqn (7) to our conditions, PS has *n* ≈ 100 ≫ 1 at its limiting *M*_W_ of 10 000 g mol^−1^ in mechanical degradation. We now write *K*_*n*_ as *K*_0_, and equate it to the monomer activity *a*_m_, leading to a simple relationship between monomer activity *a*_m_ and the Gibbs energy of polymerization Δ_r_*G*:8
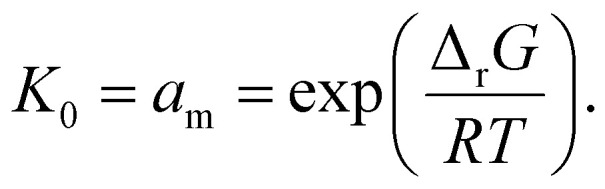


At this point we may use eqn (8) directly to plot the depropagation equilibrium constants for PE, PP and PS as functions of *T*, which can be termed the local temperature at which depropagation occurs during grinding impacts. This is not necessarily the temperature at which chain radicals are generated, but rather the temperature at which reactions 3 and 4 in Fig. 2 may occur from preexisting radicals. These equilibrium constants as a function of temperature are depicted in Fig. 3a. The results do conform to the thermodynamic viability of depolymerization of the polymers, with PS > PP > PE for depolymerization to gaseous monomer according to their ceiling temperatures.^7^

**Fig. 3:**
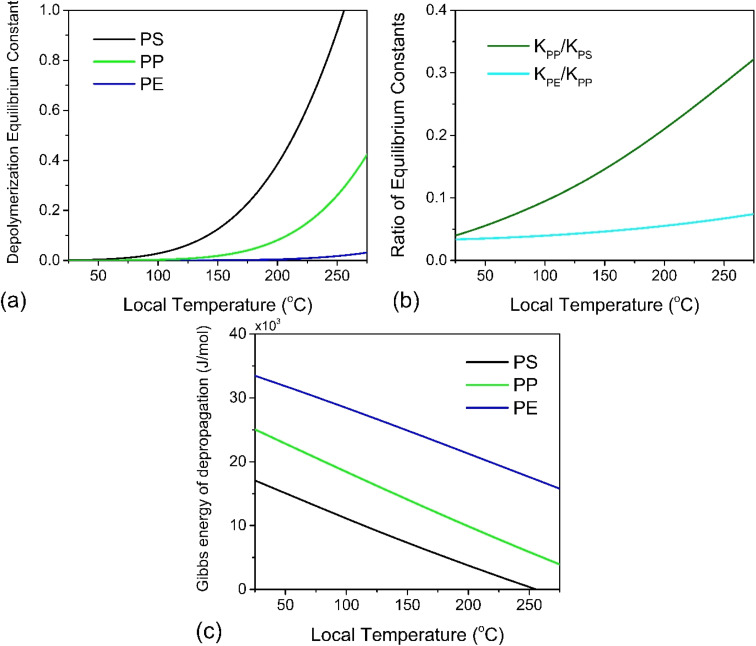
(a) Depropagation equilibrium constants of PS, PP and PE to gaseous state monomer as a function of hot spot temperature, (b) ratio of these constants for PP over PS and for PE over PP as a function of hot spot temperature, (c) Gibbs energy of depropagation for PS, PP, PE to gaseous monomer.

In Fig. 3b we also plot the ratio of equilibrium constants for PP over PS and for PE over PP over the studied hot spot *T* range, which illustrates an order of magnitude difference in reactivity between PS and PP, and the same between PP and PE. Because the temperature range for which thermodynamic data has been tabulated terminates around the ceiling temperature of PS and well below that of PP and PE, the equilibrium constants all fall below the value of 1. To achieve a high conversion in reactions like these one can increase the process temperature or remove enough of the product, so that the forward reaction can continue without entirely reaching equilibrium.

The quantity *a*_m_ in eqn (8) can be linked to a controllable system variable, namely the gas phase monomer partial pressure *p*_m_, through the fugacity coefficient *η*_m_ and the standard pressure *p*_0_:9
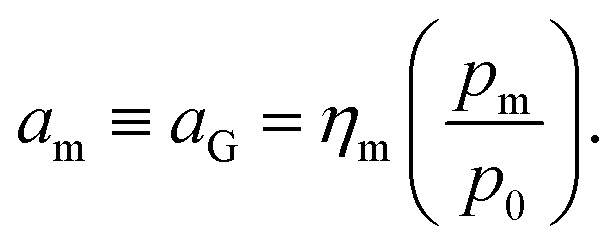


*η*_m_ may be calculated using thermodynamics simulation software for a homogeneous unreactive gas mixture closed system consisting of various concentrations of monomer together with pure nitrogen, at various temperatures and pressures. Reflecting the practical conditions in the laboratory scale ball mill, the parameter space to simulate *η*_m_ was chosen as follows: temperature range of 273–403 K at 1 atm, pressure range of 1–5 atm at 298 K, monomer molar fraction range of 0.005–0.5. Regardless, it was found that for ethylene, propylene and styrene, *η*_m_ is nearly unity across the range of conditions simulated – changing insignificantly with gas phase temperature or pressure. This means the plots of monomer pressure fraction *p*_m_/*p*_0_ as a function of the hot spot temperature is nearly identical to the *K*_0_ = *a*_G_ curves in Fig. 3a.

The results depicted in Fig. 3 have important implications regarding the achievable extent of depolymerization. Since mechanochemical depolymerization occurs at macroscopic temperatures well below the ceiling temperature of poly(olefin)s and the reaction is endothermic, there must be a transfer of the kinetic energy of the mill to the polymer to drive the depolymerization reaction. Of the two leading mechanisms of energy transfer postulated for mechanochemical environments, the first is the so-called “hot spot” mechanism,^21^ which involves kinetic energy of the grinding action being transformed into thermal energy characterized by a change in local temperature. This thermal energy is in turn absorbed by the polymer in the endothermic depropagation reaction, and thus, depolymerization relies on heat as driving force. Applying this mechanism to our system, suppose that the activated volume in which a depropagation occurs is characterized by a length scale *λ* which is lower-bounded to be on the order of molecular dimensions (nanometers). The depropagation reaction of a poly(olefin) is characterized by a positive enthalpy of reaction Δ_r_*H*°. Thus, an amount of energy given by Δ_r_*H*°/*N*_A_ is absorbed by every depropagation event, where *N*_A_ is Avogadro's constant. The activated volume of the polymer *λ*^3^ must contain enough transferable energy for this step to happen, and in the hot spot mechanism, this energy is in the form of heat generated by friction and plastic deformation during impact that is situated in or near the activated volume. If the polymer material has heat capacity *C*_p_ and density *ρ* which are functions of temperature, we may calculate a temperature change Δ*T* associated with the activated volume *λ*^3^ of polymer where a depropagation reaction occurs, by solving the following equation for Δ*T*:10
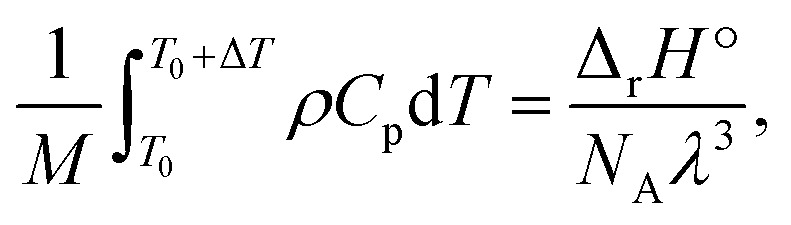
where *T*_0_ is the initial temperature of the activated volume, and *M* is the molar mass of the monomer. We apply this thought experiment to PS as the illustrative example due to its readily available heat capacity^42^ and density^45^ data across a wide temperature range. For PS, Δ_r_*H*° = 41 000 J mol^−1^ (for depolymerization) at *T*_0_ = 298 K.^43^ Solving eqn (10) for Δ*T* with various values of *λ* in the nanometer range results in Fig. 4a, which illustrates the inverse relation between these two properties. For the minimum realistic activated volume of 1 nm^3^ of PS to generate a monomer, this corresponds to a temperature decrease of around 50 °C in that volume. If the temperature of a hot spot is reduced from 200 °C to 150 °C the equilibrium constant for the depropagation reaction in the activated volume decreases from 0.4 to less than 0.2 according to the curve for PS in Fig. 3a. This suggests that polymer particles inside the reactor experience hot spot temperatures for only brief moments leading up to a depropagation event that consumes most of that heat. Consequently, engineering the amount of heat that is available in each hot spot appears to be one of the most important design criteria for mechanochemical depolymerization processes.

**Fig. 4:**
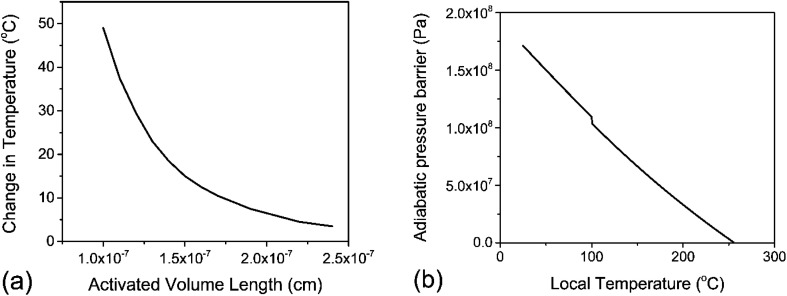
PS depropagation proceeding along mostly thermal or mostly adiabatic compression mechanisms; (a) solution to eqn (10) – activated volume length *λ versus* change in temperature Δ*T* within the volume *λ*^3^ assuming isothermal depropagation, (b) energy barrier for depropagation in the adiabatic compression mechanism expressed as a pressure (energy density) *versus* temperature.

It is recognized that the energy of an activated domain in a mechanochemical process may be in a form that is distinct from heat.^46^ However, similar arguments about thermodynamic limits of the depropagation reaction can be made in this case. Notably, Zhurkov *et al.* showed that the driving forces of certain mechanochemical reactions of polymers can be described as a distorting/straining of bonds resulting in a reduced activation energy. This is the widely accepted mechanism of mechanochemical chain scission (reaction 1 in Fig. 2),^35,47,48^ and from the perspective of thermodynamic energy transfer it can be regarded as the direct absorption of kinetic energy by the polymer chain which becomes chemical energy – an ideally adiabatic process. Considering the generally short time scale of mechanochemical collisions – on the order of microseconds, notwithstanding plastic deformation of polymer particles within the impact volume which necessarily generates heat on the material scale, the actual depropagation at the molecular level may plausibly proceed adiabatically just like with the chain scission reaction. The primary uncertainty in applying the same model to depropagation reactions of terminal radicals is that strain or distortion of bonds should be largely alleviated after chain scission due to the additional degrees of freedom available to a chain end compared to a midchain segment. Nonetheless, it is worth reasoning through a scenario in which adiabatic compression could play a role similar to heat in driving mechanochemical depolymerization.

For a thermal process, we can benchmark the energetic requirements of depropagation by plotting the Gibbs energy of depropagation Δ_r_*G* calculated using eqn (2) directly as a function of temperature (Fig. 3c). In an adiabatic compression-driven process, a depropagation event can occur at a given temperature when a mechanochemical collision delivers at least the Δ_r_*G*-equivalent amount of kinetic energy to the portion of polymer being subjected to the event. The free energies in Fig. 3c can be converted from the unit of Joule per mole of monomer equivalent of the polymer to a “pressure barrier” (the unit of Pa) that needs to be overcome for the depropagation reaction to occur in the activated volume. This is expressed as 
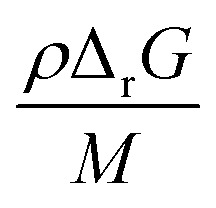
, where *ρ* is the density of the polymer as a function of temperature, and *M* is the molar mass of the monomer. Using PS again as the illustrative example, we plot 
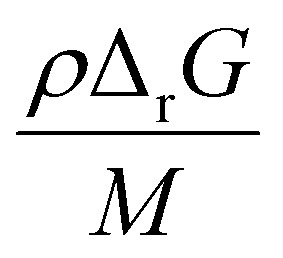
 as a function of *T* in Fig. 4b. A step change in the curve near 100 °C is due to the discontinuity in mass density near the glass transition of PS. At ambient conditions the energy density barrier is on the order of 1 × 10^8^ Pa. In a vibratory ball mill operating at 30 Hz, a 2.0 cm diameter ball can generate 7–8 × 10^8^ Pa of impact pressure,^21^ enough to overcome this pressure barrier during the collision, but decompression will likely occur in fractions of a millisecond limiting the time for consecutive reactions to occur. This result indicates that the conversion of PS is just thermodynamically feasible enough to make mechanochemical depolymerization viable near ambient conditions. Meanwhile, it is apparent from Fig. 3c that 
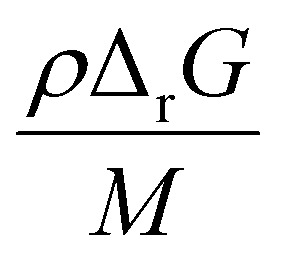
 for PP is several times greater than for PS, and that the value for PE is about an order of magnitude larger. Hence, PP can be regarded as just outside the thermodynamically viable range – and PE even more so – when milled under the same conditions as PS. Increasing the energy of collisions is therefore an unambiguous goal when depolymerizing less reactive poly(olefin)s.

Experimental data with PS indicate that appreciable levels of monomer were detected in both the reactor and the effluent gas, which implies a partitioning of monomer between the distinct phases of the system as they are produced.^27^ The assumption that all monomers generated during milling are gaseous cannot explain why monomers were detected associated with the solid phase after milling. To account for this observation, we propose that some quantity of gaseous monomer fluid produced by transient depolymerization events does not partition into the bulk gas phase upon formation but is instead situated in the solid polymer matrix of the particle or adsorbed on its surface at temperature *T*_P_, where it is likely to be reabsorbed by the polymer through the propagation step (4 in Fig. 1) by radicals in the interior of the particle, helped along by the tendency of olefinic monomers to dissolve in their bulk polymer.^49^ Significant repolymerization was indeed observed in the ball milling of PS in a sealed reactor where generated monomer accumulated,^27^ which verifies that a competing driving force is present. To maximize depolymerization yield, the competing driving force should be suppressed and it is thus important to gain an understanding of its significance for each polymer under consideration.

To describe this phenomenon thermodynamically, we assume that all particles on the reactor time scale (when not participating in collisions) have the same temperature *T*_P_ and the amount of monomer fluid in the reactor at steady state conditions is distributed evenly across all particles with activity *a*_P_. This is a reasonable assumption given the vigorous mixing conditions in the ball mill which guarantee that, statistically, all particles experience about the same rate of impact under sustained milling, and the particles spend most of the time in a resting state. From the “wet” polymer matrix, monomers may volatilize continuously into the turbulent gas phase flowing past the particle surface, as depicted in Fig. 2. Consider the following two-stage equilibrium with associated equilibrium constants:11
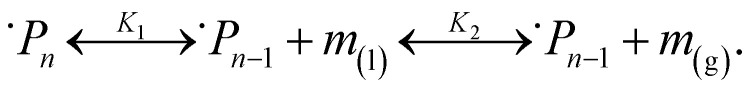
12
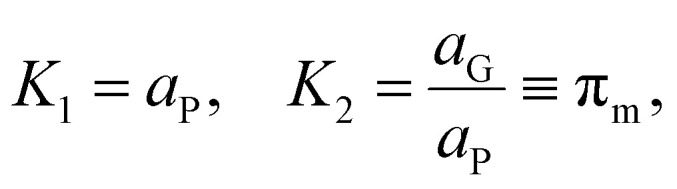
where we explicitly notate *m*_(l)_ as the monomer species in the “liquid” phase within the polymer particle and *m*_(g)_ as the same monomer species in the gas phase. Due to the assumption introduced in eqn (8), we omitted the polymer radical species activities from the equilibrium constants, and associated *K*_2_ specifically with a thermodynamic partition coefficient *π*_m_, the ratio of *a*_G_ to *a*_P_.

Combining the two equilibria depicted in eqn (11) with the earlier equilibrium introduced in eqn (8), which is based directly on thermodynamic data, we obtain the following expression for *K*_1_:13
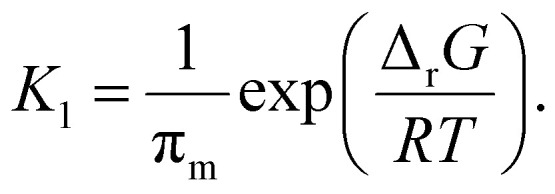


It is the hot spot temperature *T* that appears in eqn (13) because the logic of our model dictates that the solid–gas equilibrium between chain radical and monomer at temperature *T* ultimately determines the amount of monomers available for phase partitioning. If *π*_m_ is computed, we obtain the characteristic *K*_1_ as a function of hot spot temperature through substitution of eqn (9) and (10) for *a*_G_, which can be interpreted as the tendency of a monomer type to remain in the particle phase after its generation from a live polymer radical. The tendency of monomer to dissolve in its own polymer is described analytically using Flory–Huggins theory,^50,51^ but owing to the low quantity of monomer (<15 mg g^−1^ in the case of PS)^27^ partitioned to the particle phase at steady state, the precise properties of the particle phase was approximated as a heavy hydrocarbon fluid out of convenience.

Using a closed two-phase system with a nitrogen gas phase, a temperature range of 298–398 K and a total monomer concentration of 10^−4^ to 5 mol L^−1^ at standard pressure, *π*_m_ was simulated. A plot of *K*_1_*versus* hot spot temperature for PE, PP and PS is depicted in Fig. 5a–c. The results conform to the expected behavior of the respective monomers of these three polymers. The partition coefficient increases with increasing particle temperature, which leads to more monomers partitioning to the gas phase. However, the magnitude of this effect differs by almost four orders of magnitude between PS and PP, and two orders between PP and PE, which is readily apparent by plotting the ratios of these liquid–side equilibrium constants for PP to PS, and PE to PP, as was done in Fig. 5d and e. Styrene exhibits the lowest partition coefficient among the monomers (*π*_m_ < 1 for all simulated conditions, see Fig. 5f), and the magnitude of *K*_1_ indicates that a significant quantity will always remain associated with the particle. This tendency also serves as a natural corollary to the repolymerization documented for PS depolymerization in a sealed ball mill reactor. The fugacity coefficient *η*_m_ of styrene is insensitive to temperature but decreases slowly with increasing gas phase pressure, which indicates that if monomer recovery is to be maximized with *K*_0_ ≫ *K*_1_, a high-pressure flow setup would be recommendable for PS depolymerization in a ball mill, though this may lead to additional downstream separation costs. For PP however, *K*_0_ ≫ *K*_1_ is guaranteed automatically by the volatility of propylene, and this is even more true of PE. In fact, for these two poly(olefin)s, it can be concluded that any monomer produced inside the ball mill at any set of conditions will have a high probability of exiting the reactor in the effluent gas stream. The real challenge is instead in the low intrinsic value of *K*_0_ – the difference in reactivity differs by multiple orders of magnitudes across these three polymers. Even without considering the greater instability of chain end radicals in PE and PP, these results suggest that depolymerization chemistries other than thermochemical depropagation – such as oxidation or hydrogenation^7^ – are promising strategies towards achieving solid-state depolymerization of these plastics.

**Fig. 5:**
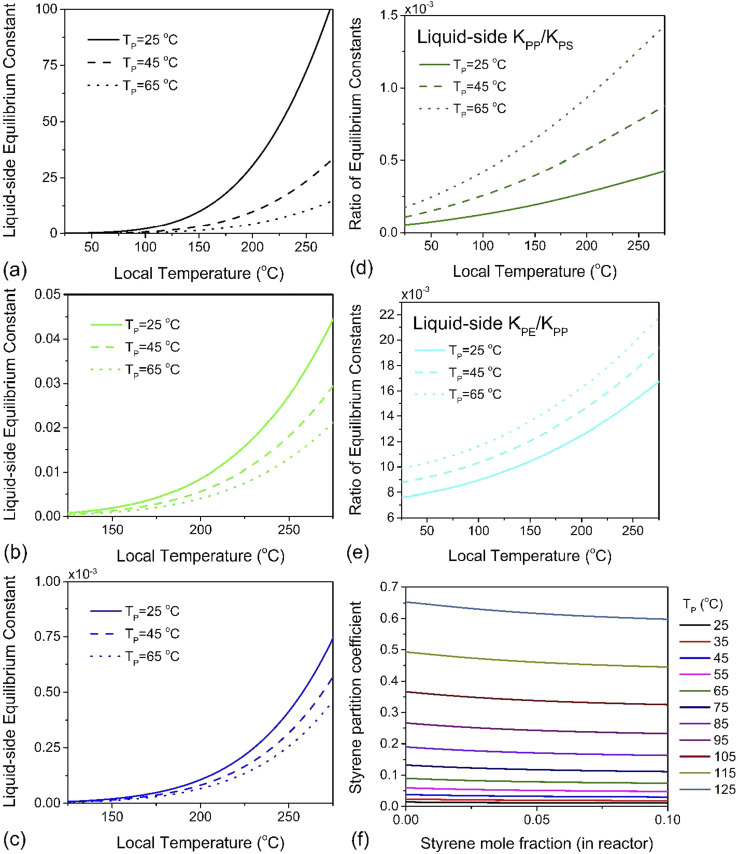
The liquid-side equilibrium constant *K*_1_ as a function of depolymerization temperature for (a) PS, (b) PP and (c) PE at average particle temperatures of *T*_P_ = 298 K, 318 K and 338 K, and at each of these temperatures, the ratio of *K*_1_'s (d) for PP over PS and (e) for PE over PP. (f) Partition coefficient *π*_m_ of styrene defined in eqn (12) as a function of styrene mole fraction for various PS particle temperatures.

In conclusion, an analysis of the thermodynamics of mechanochemical depolymerization of PE, PP and PS shows that these reactions are typically thermodynamically limited. Rather than the availability of radicals, the amount of energy in activated volumes appears to limit the extent of the endothermic depropagation reaction under typical milling conditions. Thus, the creation of sufficiently energy-rich domains is the most critical challenge for developing mechanochemical plastics recycling processes. Specifically, hotter, larger, and longer-lived hotspots (or otherwise excited domains) are desirable. Alternatively, depolymerization can be coupled with hydrogenation or oxidation of the fragments to make the reaction much more favorable even under very mild conditions.^7,25,52^

Thermodynamically limited depolymerization reactions can also be promoted by effective product removal, but the physical properties of the monomers need to be accounted for. PS (and by extension PMMA) can depolymerize appreciably based purely on its thermodynamic properties if energy-dense activated volumes are generated consistently inside the ball mill environment, but a significant fraction of the products remains associated with residual polymer. On the other hand, the monomers of PP and PE do not have any significant phase partitioning barriers, but their equilibrium constants of depolymerization are also many times lower than that of PS at the same temperature, which results in depolymerization kinetics that are orders of magnitude slower than those of PS.

## Data availability

Literature data used in this article, including heat capacity *versus* temperature relationships for PE, PP and PS and the density *versus* temperature relationship for PS are available from entries 40–43 and 45 in the References. The results featured in the figures of this article derived from the literature data are tabulated in the ESI.†

## Conflicts of interest

There are no conflicts to declare.

## Supplementary Material

MR-001-D4MR00079J-s001
